# Role of Therapeutic Plasma Exchange in Accelerating the Clearance of Perfluoroalkyl and Polyfluoroalkyl Substances

**DOI:** 10.7759/cureus.116975

**Published:** 2026-09-26

**Authors:** Yigit Baykara

**Affiliations:** 1 Pathology, Banner - University Medical Center Tucson, Tucson, USA

**Keywords:** apheresis, perfluoroalkyl substances, pfas, polyfluoroalkyl substances (pfas), therapeutic plasma exchange

## Abstract

Per- and polyfluoroalkyl substances (PFAS) are persistent synthetic compounds linked to adverse health outcomes. PFAS bind strongly to serum albumin and, combined with continuous renal reabsorption and enterohepatic recirculation, prevent efficient excretion and extend their biological half-life to several years. Because PFAS remain largely confined to the intravascular compartment, the targeted removal of the plasma fraction offers a viable clearance mechanism. Recent clinical evidence, including a randomized controlled trial in occupationally exposed firefighters, demonstrates that regular plasma and whole blood donations significantly reduce serum PFAS burdens. Furthermore, double filtration plasmapheresis (DFPP) also achieved significant clearance of serum perfluorooctane sulfonic acid (PFOS) levels. Since the removal of plasma is effective in the reduction of serum levels of PFAS, therapeutic plasma exchange (TPE) may be a beneficial intervention that is capable of accelerating the clearance of these "forever chemicals" and mitigating systemic toxic burdens in high-risk cohorts.

## Editorial

Introduction

Per- and polyfluoroalkyl substances (PFAS) represent a broad category of synthetic fluorinated organic compounds widely utilized in industrial and consumer applications. Often termed "forever chemicals," their carbon-fluorine bonds resist environmental and metabolic degradation [1]. Therefore, they accumulate in human tissues over time. The primary challenge in managing PFAS toxicity lies in their unique toxicokinetics. Unlike classic lipophilic persistent organic pollutants that sequester predominantly in adipose tissue, PFAS exhibit a high affinity for serum proteins, particularly albumin [2,3]. This protein-binding property, combined with renal reabsorption and enterohepatic recirculation [4], extends the human biological half-life of legacy compounds such as perfluorooctane sulfonic acid (PFOS) and perfluorooctanoic acid (PFOA) to several years [3,5]. Given the epidemiological associations between chronic PFAS exposure and metabolic, cardiovascular, reproductive, and immune adverse outcomes, identifying clinical interventions capable of clearing these substances is essential [6,7].

Mechanisms of PFAS persistence and clearance obstacles

To understand why standard clearance mechanisms fail, it is necessary to evaluate how PFAS behave intravascularly. Once absorbed, long-chain PFAS bind strongly to the fatty acid-binding pockets of serum albumin [8]. Because they remain confined mostly to the intravascular and extracellular compartments rather than fat stores, their elimination is governed by the liver and kidneys.

While a portion of circulating PFAS is secreted into bile, the vast majority undergoes highly efficient reabsorption in the distal gastrointestinal tract via enterohepatic pathways [4]. Similarly, filtered PFAS in the renal glomerulus are extensively reabsorbed by organic anion transporters in the proximal tubules [4]. This continuous internal recycling loop minimizes fecal and urinary excretion. Interventions must therefore either disrupt this reabsorption or physically extract the protein-bound fraction from the intravascular compartment.

Clinical evidence for plasma and whole blood donation

The concept of utilizing apheresis to lower human PFAS burdens was pioneered in early clinical case studies by Genuis et al., who suggested significantly accelerated perfluoroalkyl acid clearance with regular phlebotomy treatment [9]. However, the most structured clinical validation of plasma separation techniques for PFAS reduction comes from a randomized controlled trial involving occupational cohorts [10]. Firefighters exhibit significantly elevated serum PFAS levels, which correlate with an increased occupational risk of adverse health outcomes [11]. Gasiorowski et al. evaluated the efficacy of regular plasma and whole blood donation versus observation over a 12-month period in a cohort of Australian firefighters, a population disproportionately exposed to PFAS via aqueous film-forming foams [10]. They showed that plasma and whole blood donations caused greater reductions in serum PFOS levels than observation alone and greater reductions in serum perfluorohexane sulfonic acid levels by plasma donation, while no significant change was observed in the whole blood donation or observation groups. The removal of plasma and albumin-bound PFAS with it decreases serum PFAS levels by effectively bypassing renal reabsorption and enterohepatic circulation.

Clinical evidence for therapeutic plasma exchange (TPE)

While hemodialysis removes small and water-soluble molecules such as toxins and electrolytes, therapeutic plasma exchange (TPE) removes large and high-molecular-weight molecules such as plasma proteins or protein-bound substances. Liu et al. demonstrated the expanded utility of double filtration plasmapheresis (DFPP) utilizing a polyvinyl alcohol-based membrane [12]. In their study, they enrolled patients with hyperlipidemia who were medically refractory or did not tolerate the medications. In addition to the post-pheresis lipid profile, they also evaluated the inflammatory markers and the environmental toxins PFOA and PFOS. DFPP not only optimized the patients' lipid profiles and lowered systemic serum inflammation but also achieved significant clearance of serum PFOS levels. These findings suggest that TPE can potentially cause greater reductions in serum PFAS levels in a short period of time, as it allows for the removal of larger volumes of plasma compared to plasma donation.

Clinical considerations and future directions

From a clinical standpoint, implementing apheresis for environmental toxin clearance requires careful patient selection. It is not a generalized public health screening tool but rather a targeted intervention for individuals with documented, critically high occupational or accidental exposure profiles (e.g., firefighters, chemical manufacturing workers, or heavily contaminated communities). Before widespread implementation can be recommended, several gaps must be addressed. Following a rapid drop in serum PFAS levels post-apheresis, a rebound effect would typically occur as tissue-bound stores gradually mobilize back into the intravascular compartment. Moreover, the replacement fluid used, number of treatments, and optimal frequency to achieve sustained systemic depletion should be defined. Since PFAS bind avidly to albumin, exchanging the patient's PFAS-laden plasma for PFAS-free albumin could, in principle, enhance a "sink condition" that promotes the redistribution of PFAS from tissue reservoirs back into circulation. However, while reducing the absolute serum PFAS levels would be a clear technical success, studies must show that decreased serum levels translate into a measurable reduction in clinical disease incidence.

Conclusion

TPE might be a beneficial intervention that is capable of accelerating the clearance of these "forever chemicals" and mitigating systemic toxic burdens in high-risk cohorts such as firefighters and chemical manufacturing workers, as it can remove large, protein-bound molecules by effectively bypassing renal reabsorption and enterohepatic circulation. However, the current clinical evidence is limited and based on plasma and whole blood donations and DFPP rather than conventional TPE. Future studies using conventional TPE are needed to define optimal session intervals, evaluate the long-term benefit-to-risk ratio and the cost-effectiveness of repeated plasma exchange, and verify whether decreased serum PFAS levels translate into measurable reductions in cardiovascular, immunological, reproductive, and metabolic adverse effects before translating TPE into routine clinical practice.
